# A flotation/sieving method to detect *Echinococcus multilocularis* and *Toxocara* spp. eggs in soil by real-time PCR

**DOI:** 10.1051/parasite/2017029

**Published:** 2017-07-24

**Authors:** Gérald Umhang, Matthieu Bastien, Camille Renault, Marine Faisse, Christophe Caillot, Jean-Marc Boucher, Vanessa Hormaz, Marie-Lazarine Poulle, Franck Boué

**Affiliations:** 1 ANSES Nancy Laboratory for Rabies and Wildlife, National Reference Laboratory for Echinococcus spp., Wildlife Surveillance and Eco-Epidemiology Unit, Technopole Agricole et Vétérinaire 54220 Malzéville France; 2 University of Reims Champagne-Ardenne, SFR Cap Santé, EA 3800 PROTAL 51092 Reims cedex France; 3 University of Reims Champagne-Ardenne, CERFE 08240 Boult-aux-Bois France; 4 French Establishment for Fighting Zoonoses (ELIZ), Domaine de Pixerécourt 54220 Malzéville France

**Keywords:** Environmental samples, Soil contamination, *Echinococcus multilocularis*, *Toxocara* spp., Parasite eggs, Real-time PCR

## Abstract

Soil can be a source of human infection by many zoonotic helminth species including *Echinococcus multilocularis* and *Toxocara* spp. The prevention of alveolar echinococcosis could be greatly improved through the identification of at-risk areas. Yet very few data are available about the detection of *E. multilocularis* in soil, while more studies have been reported for *Toxocara* spp. Identification of soil contamination by *E. multilocularis* eggs requires the use of specific methods. This study describes the development of a method for the detection of *E. multilocularis* in soil samples with the concentration of eggs using a flotation/sieving method and detection by duplex real-time polymerase chain reaction (PCR). *Toxocara* spp. egg detection was also undertaken due to the widespread presence of this parasite in soil, despite it being considered less pathogenic. Method sensitivity of 100% was reached for the detection of 10 *E. multilocularis* eggs spiked in 10 g of soil. Concerning *Toxocara* spp., method sensitivity was lower but assumed to be due to the reduced effectiveness of the DNA extraction protocol. The parasitological status for *E. multilocularis* and *Toxocara* spp. of 63 carnivore fecal samples collected in highly endemic rural areas of France and of soil samples collected under and near these fecal samples was compared. The contamination of soil samples collected under positive fecal samples for *E. multilocularis* (*n* = 3) or *Toxocara* spp. (*n* = 19) confirmed the transfer of eggs from the definitive host to the environment.

## Abstract (fr)

Le sol peut être une source d’infection humaine par de nombreuses espèces d’helminthes zoonotiques, dont *Echinococcus multilocularis* et *Toxocara* spp. La prévention de l’échinococcose alvéolaire pourrait être grandement améliorée par l’identification des zones à risque. Pourtant, très peu de données sont disponibles sur la détection d’*E. multilocularis* dans le sol, alors que d’autres études ont été rapportées pour *Toxocara* spp. L’identification de la contamination des sols par les œufs d’*E. multilocularis* nécessite l’utilisation de méthodes spécifiques. Cette étude décrit le développement d’une méthode pour la détection d’*E. multilocularis* dans les échantillons de sols avec la concentration d’oeufs en utilisant une méthode de flottation / tamisage et la détection par PCR duplex en temps réel. La détection des œufs de *Toxocara* spp. a également été réalisée en raison de la présence généralisée de ce parasite dans le sol, bien qu’il soit considéré comme moins pathogène. Une sensibilité méthodique de 100 % a été atteinte pour la détection de dix oeufs d’*E. multilocularis* mélangés à dix grammes de sol. En ce qui concerne *Toxocara* spp., la sensibilité méthodique était inférieure mais nous supposons que cela était dû à l’efficacité réduite du protocole d’extraction d’ADN. Le statut parasitologique pour *E. multilocularis* et *Toxocara* spp. de 63 échantillons de fèces de carnivores recueillis dans des zones rurales fortement endémiques de France a été comparé à celui d’échantillons de sol prélevés en dessous ou à proximité de ces fèces. La contamination des échantillons de sol recueillis sous les échantillons fécaux positifs pour *E. multilocularis* (*n* = 3) ou *Toxocara* spp. (*n* = 19) a confirmé le transfert des œufs de l’hôte définitif vers l’environnement.

## Introduction

More than 1.5 billion people (24% of the world’s population) are infected with soil-transmitted helminths worldwide [27]. Echinococcosis, trichinellosis, and toxoplasmosis are the main parasitic diseases of concern in Europe according to the European Food Safety Authority [17]. Among *Echinococcus* species, *Echinococcus multilocularis*, causing alveolar echinococcosis, is currently a real threat to public health in Europe, with a larger endemic area than previously thought [7, 24, 30]. Alveolar echinococcosis is caused by the oral ingestion of microscopic eggs developing into the larval stage of the tapeworm. The metacestode is made up of small chains of interconnected vesicles almost exclusively in the liver, with tumor-like, infiltrative, destructive growth [4, 11]. Humans are considered as accidental hosts of the parasite. Its lifecycle in Europe is mainly sylvatic involving red foxes (*Vulpes vulpes*) as definitive hosts, and small rodents as intermediate hosts. Nevertheless, concerning domestic carnivores, cats (*Felis silvestris catus*) and especially dogs (*Canis lupus domesticus*) can also act as definitive hosts after predation of infected rodents. Cats are not considered to be particularly significant due to the very low number of eggs produced during their patent period [12]. The development of worms in the intestines of definitive hosts results in the production and release of eggs to the soil *via* feces. Worm burden in foxes is known to be very heterogeneous with few foxes harboring the vast majority of worms, leading to a heterogeneous distribution of contaminated feces responsible for environmental contamination [3, 8, 29]. In humans, the long incubation period (from 5 to 15 years) makes the identification of the source of infection difficult or impossible. Some recurrent potential risk factors for developing alveolar echinococcosis in Europe have been identified as “dog or cat ownership,” “living in a rural area,” “having a kitchen garden,” “farming,” and “handling foxes” [13, 16, 26]. While humans can be infected through direct contact with infected carnivores having eggs on their fur or their feces, environmental contamination (soil, water, vegetables, and fruits) is thought to represent another source of infection. Many surveillance studies have been conducted in highly *E. multilocularis* endemic countries in Europe. These aimed to establish the prevalence of the parasite in different host species from animal samples (intestines, feces, or liver). These data provide information on the overall presence of the parasite in the different investigated areas but are not useful in identifying the source of risk for alveolar echinococcosis in humans. As feces are the primary source of infective eggs, data obtained from feces samples may constitute a proxy for describing environmental contamination by the parasite. In addition, the examination of soil contamination by *E. multilocularis* eggs to identify at-risk areas would result in a better understanding of the source of human infection and associated risk factors. Nevertheless, detection of taeniid eggs in feces suffers from low sensitivity by conventional routine diagnosis. The enrichment of taeniid eggs and their subsequent analysis by Polymerase Chain Reaction (PCR) by the flotation and sieving method as per Mathis et al. [21] has overcome the low sensitivity observed in conventional routine diagnosis, and opened new diagnostic strategies [2]. Difficulties related to the environmental samples may explain why only very few recent studies have focused on the identification of *E. multilocularis* eggs in environmental samples such as soil, vegetables, and fruits [5, 18, 22, 31]. These studies have provided preliminary results that need to be confirmed by additional data.

In this context, the aim of this study was to develop a flotation/sieving method for the detection of *E. multilocularis* eggs in soil samples by real-time PCR, with evaluation of method sensitivity. Among the many other helminth eggs that may be found in soil, *Toxocara* spp. has a lifecycle also based on the release of eggs *via* carnivore feces to the soil and ubiquitous presence. This nematode genus is responsible for *larva migrans* in humans, mainly infected by environmental sources [6], and constitutes a public health problem [25]. The detection of this zoonotic parasite was thus also evaluated with the method first established for *E. multilocularis*. To illustrate the utilization of this method, soil samples collected under and near carnivore fecal samples were tested in order to improve our understanding of the transfer of eggs from hosts to the environment.

## Materials and methods

### Origins of soil samples and *E. multilocularis* and *Toxocara* spp. eggs used to develop the method

The soil used for the development of the method was sampled at the experimental station of the ANSES laboratory. No free-ranging carnivores have access to this area, allowing for the collection of soil samples free from helminth eggs. *E. multilocularis* eggs were obtained from a fecal sample removed from the colon of a naturally infected fox diagnosed by the sedimentation and counting technique (SCT) exhibiting no worms of the *Taenia* genus [29]. The fecal sample was frozen at −20 °C until its use for isolating eggs. Additionally, the exclusive presence of *E. multilocularis* eggs was confirmed by PCR by the individual testing of 20 eggs randomly sampled and all confirmed to be *E. multilocularis*. Concerning *Toxocara* spp., embryonated eggs came from a fecal sample collected on the ground in the department of Moselle from a naturally infected cat. The diagnosis of *Toxocara* spp. was performed by real-time PCR [15] and the fecal samples frozen at −20 °C until use. For both parasites, fecal samples were homogenized in distilled water and then filtered through a 120 μm nylon mesh. After centrifugation, the supernatant was discarded and the pellet stored at 4 °C until it was suspended and observed under a stereoscopic microscope (Olympus SZX16, 250× magnification). A home-made micropipette prepared from a streamlined heparinized capillary tube (select) was used to collect the eggs one by one. The soil samples spiked with parasite eggs were stored at 4 °C for a few hours before being analyzed.

### Concentration of helminth eggs from the soil samples

The flotation method involved using 10 g soil samples either spiked with *E. multilocularis* or *Toxocara* spp. eggs or samples directly collected in the environment. Samples were mixed with 10 mL of a 0.2% solution of Tween 20 in conical centrifuge tubes in order to facilitate the separation of eggs from soil particles. After centrifugation (1000 *g*, 15 min), the supernatant was discarded. The pellet was suspended by mixing with 15 mL of a zinc chloride solution with a specific density of 1.42. After centrifugation (1000 *g*, 20 min), the supernatant was transferred to be filtered on a 20 μm nylon mesh (Buisine) using a suction pump. The substrate in the mesh was then rinsed with 50 mL of a 0.2% Tween 20 solution above a funnel placed on a centrifugation tube. A new mesh was used for each sample. After new centrifugation (1000 *g*, 15 min), the supernatant was discarded and a pellet of approximately 1 mL retained to undergo DNA extraction. In the context of the method’s development, samples of 20 g of soil were also tested for *E. multilocularis*. The same flotation protocol was applied but volumes were doubled for the initial step of Tween 20 and for zinc chloride. All transfers of solution and samples were performed with new sterile pipettes to prevent any contamination between samples.

### DNA extraction and qPCR assays

DNA extraction from pellets of soil samples was undertaken using the NucleoSpin Tissue kit (Macherey-Nagel) as recommended by the manufacturer. The detection of DNA from both *E. multilocularis* and *Toxocara* spp. was undertaken by real-time PCR (qPCR) as previously described [14, 15]. The two qPCRs were performed as one multiplex reaction, also including detection of an internal control [32]. However, a different probe was used to specifically identify each source of DNA. A final qPCR volume of 25 μL was used, containing 5 μL of DNA, 12.5 μL of Master Mix Maxima Probe, 50 copies of the internal control plasmid, 0.6 μM of primers for *E. multilocularis*, 0.3 μM of primers for *Toxocara* spp., and 0.25 μM for the three probes (*E. multilocularis*, *Toxocara* spp., and internal control). The qPCRs were performed in duplicate and run on a RotorGene thermocycler (Qiagen) with a program that consists of 10 min at 95 °C and 45 cycles of 15 s at 95 °C and 60 s at 60 °C. All *E. multilocularis*-positive copro-samples obtained by real-time PCR were confirmed by sequencing the PCR products of a second real-time PCR, performed on the same gene, as proposed by Knapp et al. [15]. Briefly, the same qPCR was performed but using a new reverse primer to obtain a longer fragment. If this second qPCR is also positive, the amplicon was amplified again by a classic PCR using the same primers as the second qPCR to facilitate the sequencing. Concerning the analysis of fecal samples, 500 mg was subject to DNA extraction using the QIAamp DNA Stool Mini Kit (Qiagen), following the suggested protocols of the manufacturer. The molecular identification of the carnivore host species (i.e. fox, dog, cat) based on the fecal samples was performed as previously described [15]. Briefly, a specific couple of primers and a probe were designed for the *cob* gene of each of the three species. The qPCR was carried out in multiplex in a final volume of 25 μL, containing 2 μL of DNA, 12.5 μL of Master Mix Maxima Probe, 30 nM of Rox, 0.4 μM of forward and reverse primers, 0.1 μM of probes used for fox and dog, and 0.2 μM of probe used for cat. An Mx3005P thermocycler (Agilent) was used with a program that consists of 10 min at 95 °C and 45 cycles of 15 s at 95 °C and 60 s at 60 °C. Detection of DNA from *E. multilocularis* and *Toxocara* spp. was undertaken according to the same real-time PCR protocol described for soil samples.

### Determination of method sensitivity

The effectiveness of DNA extraction followed by detection with real-time PCRs was evaluated by testing several replicates of samples containing only distilled water with 10 eggs (*n* = 2), five eggs (*n* = 2), three eggs (*n* = 6), and one egg (*n* = 10). Secondly, the sensitivity of the method was tested throughout the process, from flotation to qPCRs, using soil samples spiked with 10 eggs, five eggs, three eggs, and one egg of both parasites. The protocol was initially designed and developed to be able to detect 10 *E. multilocularis* eggs in 10 g of soil with a minimum sensitivity of 90%. This protocol was then applied for the evaluation of sensitivity to detect eggs of *Toxocara* spp.

### Detection in soil samples under and near carnivore fecal samples

**Figure 1.**
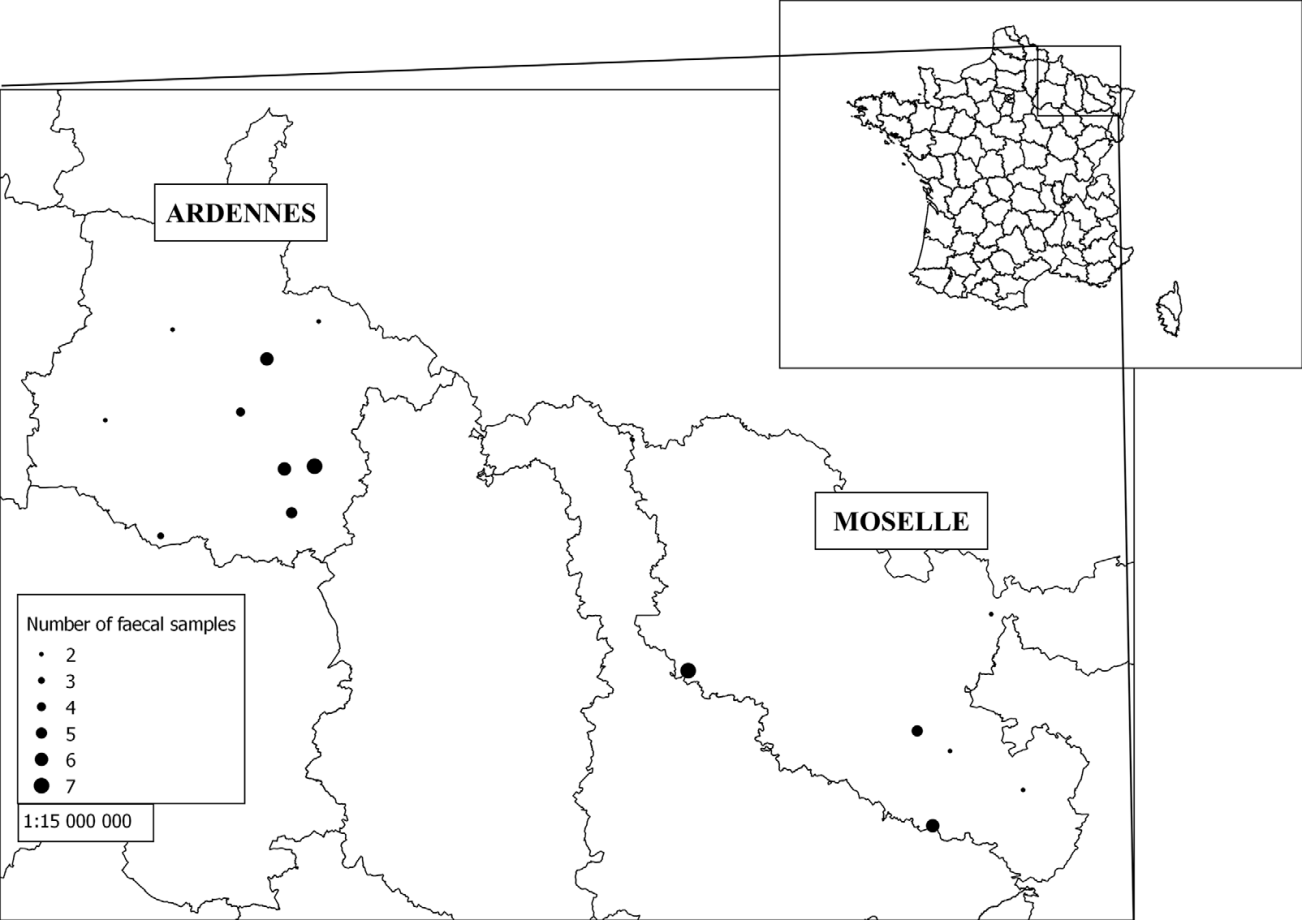
Geographical distribution of the collected feces. For each fecal sample, one soil sample was collected under the fecal sample and another one between 50 cm and 1 m from the first one.

Fecal and soil samples were collected in October–November 2015 to test the developed method. The collection was carried out at 49 sites located in two *E. multilocularis* endemic areas of north-eastern France, Ardennes and Moselle, with 36% and 34% of foxes infected, respectively [1]. One fecal sample was collected per site except for four sites where two fecal samples (*n* = 10) and three fecal samples (*n* = 2) were collected for a total of 63 carnivore fecal samples collected (Fig. 1). The carnivore origin of the fecal samples was systematically confirmed by real-time PCR. One soil sample was collected at the exact place where a fecal sample was found and a second soil sample was also taken between 50 cm and 1 m from the first one. A soil sample corresponding to roughly 50 g of soil was collected over approximately the first 5 cm of the surface, and over a surface area of approximately 10 cm^2^. The soil samples were stored frozen in a plastic zip bag. For analysis, 10 g was used after brief manual homogenization through the plastic bag. In total, two soil samples were collected for each fecal sample leading to 126 soil samples. Levels of the parasites were assessed both in the fecal and soil samples after storage for one week at −80 °C to prevent human infection. The link between the presence of fecal samples, regardless of their parasitological status or only considering positive fecal samples for *E. multilocularis* and/or *Toxocara* spp., and soil contamination was assessed using chi-square tests, or using Fisher’s exact test when there were not enough samples. All statistical analyses were performed using the statistical software program R 3.1.3 [28] (with a significance threshold of 0.05).

## Results

### Sensitivity of the method for detection of *E. multilocularis* and *Toxocara* spp. eggs

All *E. multilocularis* eggs in distilled water samples (from 10 eggs to 1 egg) directly subjected to DNA extraction and real-time PCR were detected. Average quantification cycle (Cq) values ranged from 26.43 for 10 eggs to 30.99 for one egg. A low standard deviation even for one egg (*SD* = 1.1) provided evidence of the high effectiveness and repeatability of these molecular steps. On the other hand, while all samples with 10 and five *Toxocara* spp. eggs in distilled water also led to a positive result, amplification was observed for only three out of the five samples with three eggs (60%), and for only three out of the 10 samples with one egg (30%). When testing naive soil samples of 10 g spiked with 10 *E. multilocularis* eggs, a positive qPCR signal was obtained for all samples, corresponding to 100% sensitivity (Table 1). When using five eggs to one egg, sensitivity decreased from 80% to 33.3%. Using 20 g of soil also resulted in lower sensitivity for detecting *E. multilocularis* eggs. For *Toxocara* spp., the sensitivity obtained with spiked soil samples (10 g) ranged from 41.7% for 10 eggs to only 8.3% for one egg (Table 1).

**Table 1.** Method sensitivities (in %) of the flotation/sieving method combined with DNA extraction and detection by real-time PCR for *E. multilocularis* and *Toxocara* spp. in soil samples (10 g or 20 g). For each number of eggs, 15 and 12 samples were used for *E. multilocularis* and *Toxocara* spp., respectively.

Number of eggs	*E. multilocularis*		*Toxocara* spp.
	10 g	20 g	10 g
1	33.3% (5/15)	NA	8.3% (1/12)
3	66.7% (10/15)	NA	25.0% (3/12)
5	80.0% (12/15)	73.3% (11/15)	8.3% (1/12)
10	100.0% (15/15)	86.7% (13/15)	41.7% (5/12)

### Detection of parasitic DNA under and near fecal samples

Among the 63 collected fecal samples, DNA from *Toxocara* spp. was identified in 19 samples (30.2%), mainly from cat fecal samples (Table 2). *E. multilocularis* was detected in only three fecal samples from foxes (4.8% of all collected fecal samples). Overall, *E. multilocularis* and *Toxocara* spp. were detected in 15 (11.7%) and 11 (8.6%) soil samples, respectively. *Toxocara* spp. and *E. multilocularis* were identified together only in one soil sample collected under a fox fecal sample positive for *E. multilocularis* but not for *Toxocara* spp. No inhibition was observed for fecal or soil samples. Due to the low number of fecal samples contaminated with eggs of *E. multilocularis*, the two parasites were considered together when studying links between fecal and soil sample contamination statuses (Table 3). When comparing parasite contamination in fecal and associated soil samples, a positive correlation was found only for the detection of the same parasite (i.e. *E. multilocularis* or *Toxocara* spp.) in both fecal and soil samples. Soil contamination by *Toxocara* spp. or *E. multilocularis* was not related to the presence of feces, regardless of the parasitological status of the fecal samples (*χ* ^2^ = 1.21; *p* = 0.271): positive soil was found in places where there was a scat (with or without parasites) as well as in places where there was not. Nevertheless, soil samples collected under positive fecal samples for *Toxocara* spp. or *E. multilocularis* were significantly more contaminated by the respective parasite species than the soil samples collected near these positive fecal samples (*χ* ^2^ = 5.64; *p* = 0.018). By contrast, soil samples collected under non-contaminated fecal samples were not more contaminated than soil samples collected near non-contaminated fecal samples (OR = 1.21; *p* = 0.729) (Table 4).

**Table 2.** *E. multilocularis* and *Toxocara* spp. infection level of feces collected in kitchen gardens depending on the carnivore species identified.

	Carnivore species feces identification			Total
	Fox	Cat	Dog	
*E. multilocularis*	3 (12.0%)	0	0	3 (4.8%)
*Toxocara* spp.	1 (4.0%)	17 (48.6%)	1 (33.3%)	19 (30.2%)
Number of feces	25	35	3	63

**Table 3.** Parasitological status for *E. multilocularis* and *Toxocara* spp. of fecal and soil samples depending on their position under or near the feces.

Parasitological status of fecal samples	Parasitological status of soil samples			
	Under the feces		Near the feces	
	*E. multilocularis*	*Toxocara* spp.	*E. multilocularis*	*Toxocara* spp.
Positive *E. multilocularis* (*n =* 3)	1	1	1	1
Positive *Toxocara* spp. (*n* = 19)	3	7	1	1
Negative (*n* = 41)	5	1	5	1

**Table 4.** Parasitological status (*E. multilocularis* and/or *Toxocara* spp.) of soil samples depending on where they were collected (under or near a fecal sample) and the parasitological status of the fecal sample. A positive correlation for the parasitological status was considered present only for the detection of the same parasite (i.e. *E. multilocularis* or *Toxocara* spp.) in both fecal and soil samples.

	Positive soil samples		Negative soil samples		
	Under fecal samples	Near fecal samples	Under fecal samples	Near fecal samples	
Positive fecal samples (*n* = 22)	10	4	12	18	*χ* ^2^, *p* = 0.018
Negative fecal samples (*n* = 41)	6	6	35	35	OR, *p* = 0.729

## Discussion

The flotation/sieving method combined with qPCR detection developed in this study proved to have sensitivity of 100% for the detection of 10 eggs of *E. multilocularis* in 10 g of soil sample. Additionally, it enabled the potential detection of only one egg in 10 g of soil sample. Evaluation of the method’s sensitivity appears essential before it can be used in field studies to draw relevant epidemiological conclusions. The use of 10 g of soil appeared to be more sensitive than the use of 20 g, even though there was no significant difference due to the low number of samples tested (*n* = 15). Using a higher quantity may be difficult and time-consuming for logistical reasons in the processing of the flotation/sieving technique, requiring the construction of specific in-house material [9], and may also reduce sensitivity. Collecting multiple samples of 10 g at different places may provide a better overview of contamination in an area than a single sample of a higher amount due to expected heterogeneity in the spatial distribution of the eggs. Furthermore, the relatively high level of *E. multilocularis* and *Toxocara* spp. eggs in 10 g soil samples from this study (11.7% and 8.6%, respectively) supports the use of this amount.

The method proved to be less sensitive for the detection of *Toxocara* spp. eggs compared to *E. multilocularis*. Positive amplification results were not systematically obtained after direct DNA extraction of less than five *Toxocara* spp. eggs. This step is most likely the reason for the lower sensitivity in detecting *Toxocara* spp. eggs in soil samples, rather than the flotation/sieving step as further adaptation of the DNA extraction protocol by increasing the lysis period resolved this issue. Additional tests of DNA extraction from several samples of one *Toxocara* spp. egg isolated from cat and fox fecal samples systematically resulted in a positive qPCR signal. Unfortunately, this modification could not be applied to the environmental samples tested since the protocol used was the one initially designed for *E. multilocularis*. Thus, the number of positive environmental samples for *Toxocara* spp. is probably underestimated in this study. Microscopic identification of the eggs instead of molecular diagnosis may also resolve this issue for *Toxocara* spp. While this visual identification is also possible for many other parasite genera, molecular identification is essential for taeniid eggs in order to distinguish between the *Taenia* and *Echinococcus* genera and precisely determine the species involved. Additionally, the use for soil samples of a DNA fishing method for the detection of *E. multilocularis* already developed for feces [10, 20] may improve the sensitivity of the protocol.

The method was tested with soil samples collected under and near fecal samples to assess a potential link between the detection of eggs and the observation of a fecal sample, assuming a potential transfer of eggs from the infected feces to the soil notably *via* rain washing. Due to the low number of positive fecal samples for *E. multilocularis*, data for both *E. multilocularis* and *Toxocara* spp. were considered, but assuming that the results obtained here are transposable to *E. multilocularis* alone. Based on our results, the detection of feces does not appear to be an absolute indicator of soil contamination but may only be an indicator of risk exposure to parasites. On the other hand, the more frequent contamination of soil samples collected under positive fecal samples for *E. multilocularis* or *Toxocara* spp., compared to soil samples collected near these fecal samples, confirms the transfer of eggs from the definitive host to the environment. Although this result was expected, it confirms the reliability of the developed method. Removal of feces was previously described as an important way to decrease *Toxocara* spp. egg contamination [23]. In a context of soil contamination prevention, simply removing the observed feces may not be completely effective and efforts should focus on restricting access to these sensitive areas by carnivores.

Very few data are currently available on *E. multilocularis* and other *Echinococcus* species in soil and other environmental samples. Other methods for detecting *E. multilocularis* in environmental samples, as recently undertaken in vegetables and fruits [5], need to be developed. As some reliable methods are now available, further studies are needed to evaluate actual environmental contamination in places where humans are often in contact with soil, such as kitchen gardens and public parks, and to evaluate the potential seasonal variation of this contamination. Additionally, quantitative estimation of the viability of *E. multilocularis* eggs in soil as already performed for *Toxoplasma gondii* [19] will also be of particular interest, especially considering the accumulation of eggs over time. The evaluation of *E. multilocularis* in environmental samples such as soil, vegetables, fruits, and water can improve our understanding of sources of human cases of alveolar echinococcosis.
